# Correlates of Self-Reported Sleep Duration in Middle-Aged and Elderly Koreans: from the Health Examinees Study

**DOI:** 10.1371/journal.pone.0123510

**Published:** 2015-05-01

**Authors:** Hyung-Suk Yoon, Jae Jeong Yang, Minkyo Song, Hwi-Won Lee, Sohee Han, Sang-Ah Lee, Ji-Yeob Choi, Jong-koo Lee, Daehee Kang

**Affiliations:** 1 Department of Biomedical Sciences, Seoul National University Graduate School, Seoul, Korea; 2 Department of Preventive Medicine, Seoul National University College of Medicine, Seoul, Korea; 3 Institute of Environmental Medicine, Seoul National University Medical Research Center, Seoul, Korea; 4 Department of Preventive Medicine, Kangwon National University, Kangwon-do, Korea; 5 Cancer Research Institute, Seoul National University, Seoul, Korea; 6 JW Lee Center for Global Medicine, Seoul National University College of Medicine, Seoul, Korea; 7 Department of Family Medicine, Seoul National University College of Medicine, Seoul, Korea; Queensland University of Technology, AUSTRALIA

## Abstract

Though various factors related to fluctuations in sleep duration have been identified, information remains limited regarding the correlates of short and long sleep duration among the Korean population. Thus, we investigated characteristics that could be associated with short and/or long sleep duration among middle-aged and elderly Koreans. A total of 84,094 subjects (27,717 men and 56,377 women) who participated in the Health Examinees Study were analyzed by using multinomial logistic regression models. To evaluate whether sociodemographic factors, lifestyle factors, psychological conditions, anthropometry results, and health conditions were associated with short and/or long sleep duration, odds ratios (ORs) and 95% confidence intervals (CIs) were estimated with sleep duration of 6–7 hours as the reference group, accounting for putative covariates. Regardless of sexual differences, we found that adverse behaviors and lifestyle factors including low educational attainment, unemployment, being unmarried, current smoking status, lack of exercise, having irregular meals, poor psychosocial well-being, frequent stress events, and poor self-rated health were significantly associated with abnormal sleep duration. Similarly, diabetes mellitus and depression showed positive associations with abnormal sleep duration in both men and women. Our findings suggest that low sociodemographic characteristics, adverse lifestyle factors, poor psychological conditions, and certain disease morbidities could be associated with abnormal sleep duration in middle-aged and elderly Koreans.

## Introduction

Sleep is indispensable to human survival. Evidence suggests that sleep imbalance, such as deprivation and/or fluctuations in usual sleep episodes, is significantly associated with diminished quality of life [1] while influencing adverse physical and psychological health outcomes [2–4]. Studies have reported that short (generally less than 6 hours per day) or long (generally more than 8–9 hours per day) sleep duration is related to an increased risk of chronic diseases such as obesity [5,6], hypertension [7], diabetes [8,9], and metabolic syndrome [10–12]. Furthermore, sleep imbalance is supposed to result in higher mortality rates across the lifespan [13–16].

Inadequate sleep duration is assumed to be detrimental to a person’s health and well-being. To prevent the potential harm linked to inadequate sleep duration, factors highly correlated with abnormal sleep duration should be investigated. Recently in epidemiology significant attention has been paid to how adverse patterns are induced during usual sleep episodes; thus, research examining the social and individual correlates of sleep duration continues to accumulate. Previous studies have indicated that individual characteristics, such as eating patterns [17], physical activity [18], socioeconomic status [19,20] and smoking habits [21], might be meaningful correlates of short or long sleep duration. Nevertheless, given that sleep patterns appear to be the result of complex interactions between sociocultural and individual factors [3], there is still need for studies exploring the effects of diverse contexts on sleep duration.

Evidence of the potential correlates of sleep duration has been reported, but little information exists for the Korean population. How adverse patterns are induced during usual sleep episodes in Koreans may provide valuable clues to understanding various cross-cultural aspects of sleep episodes, because sociocultural patterns that have occurred in both developed and developing countries have coexisted in Korea due to the rapid economic growth. In the present study, we conducted a cross-sectional analysis based on data from a large-scale cohort study in Korea in order to identify the sex-specific putative correlates of short or long sleep duration.

## Materials and Methods

### Ethical statements

This study is based on a large-scale genomic cohort study—the Health Examinees (HEXA) Study—which was approved by the Ethics Committee of the Korean Health and Genomic Study of the Korean National Institute of Health and the institutional review boards of all participating hospitals. Following a standardized study protocol, all study participants were prospectively recruited and voluntarily signed an informed consent form before entering the study.

### The HEXA Study

The HEXA study was launched to investigate the risk factors regarding epidemiological characteristics, genomic features, and gene-environment interactions for major chronic diseases in the Korean population. Between 2004 and 2008, a total of 85,323 participants aged 40–69 were recruited from health examination centers and training hospitals across the country. Brief information on the study protocol is summarized as follows: (1) all participants voluntarily signed an informed consent form before entering the study; (2) a survey using detailed, interview-based questionnaires was conducted to collect information on individual characteristics, including demographics, disease history, current medication use, lifetime consumption of tobacco and alcohol, dietary habits, physical activity, and reproductive history; (3) physical examinations covering anthropometric indices (height, weight, waist circumference (WC), body mass index (BMI), and body compositions) and measurement of blood pressure were performed by skilled medical staff; (4) biological samples (plasma, serum, buffy coat, blood cell, DNA, and urine) were collected and stored under stable conditions; and (5) laboratory analyses, such as liver function tests, lipid profiles, blood panels, and others were also conducted. Based on the unique health insurance system in Korea, the HEXA study enables repeated measurements on a nationwide scale; thus, this study can assess chronological changes in health status within the Korean population.

### Analytic study population

Among a total of 85,323 subjects aged 40–69 who had participated in the HEXA study, subjects who did not provide information on sleep duration (N = 1,229) were excluded from the present study. Thus, a total of 84,094 subjects including 27,717 men and 56,377 women were included in the final analysis.

### Assessment of sleep duration

As a measure of sleep quantity, sleep duration was elicited by posing the close-ended question, “On average, how many hours of sleep did you get per day during the past year (including nap times)?” Four response categories were given: <6 hours, 6–7 hours, 8–9 hours, and ≥10 hours. Using ‘6–7 hours’ (a normal sleep duration) as the reference point, other groups were assumed to represent the following: ‘less than 6 hours’ were short sleepers, ‘8–9 hours’ were moderately long sleepers, and ‘10 hours or more’ were long sleepers.

### Classification of variables

Characteristics that might be related to sleep duration were selected within five domains: sociodemographic factors (*i*.*e*., age, educational attainment, occupational classification, marital status, and menopausal status for women only), lifestyle factors (*i*.*e*., smoking status, alcohol consumption, eating habits, multi-vitamin intake, and physical activity), psychological conditions (*i*.*e*., psychosocial well-being status, stress events, and self-reported health status), and anthropometry results (*i*.*e*., BMI and WC).

#### Sociodemographic factors

Age was categorized into three groups: forties (40–49 years old), fifties (50–59 years old), and sixties (60–69 years old). Educational attainment was classified into three categories: middle school or below (*i*.*e*., no schooling, dropped out of elementary school, graduated elementary school or dropped out of middle school, and graduated middle school or dropped out of high school), high school graduate (*i*.*e*., graduated high school or technical/professional institution and dropped out of university), and college or above (*i*.*e*., graduated university or graduate school and above). Occupational status was classified according to major professions based on the Korean Standard Classification of Occupations (KSCO) derived from the International Standard Classification of Occupations (ISCO). Hierarchical occupational categories were classified into three groups: non-manual (*i*.*e*., legislators, senior officials and managers, professionals and related workers, clerks), manual (*i*.*e*., service and sale workers, skilled agricultural, forestry and fishery workers, craft and related trades workers, plant-machine operators/assemblers, and elementary occupations), and unemployed which included housewives. Marital status was defined as married or single, the latter including subjects who never married or were separated, divorced, and bereaved. Menopausal status was defined as postmenopausal women who have gone a year with no flow or premenopausal women who still experienced menstrual cycles.

#### Lifestyle factors

Smoking status was ascertained by posing the following question: “Have you smoked more than 20 packs of cigarettes (400 cigarettes) in your lifetime?” People who smoked a minimum of 400 cigarettes during their lifetime and continued to smoke were classified as current smokers; non-smokers who had never smoked in their lifetime and quitters were both classified as non-current smokers. Drinking status was also divided into two categories: current drinkers and non-current drinkers. Usual eating habits were determined by how many meals a subject had per day and were classified into two groups, having three meals a day *vs*. having irregular meals. Multi-vitamin users were defined as those who had been taking a multi-vitamin supplement more than once per week for the purpose of nutritional and/or health improvement; all others were regarded as non-users. Physical activity was assessed by posing the following question: “Do you do any sports regularly until you sweat?” Subjects who responded “yes” to the question were assigned to the regular exercise group; the respondents who answered “no” were assigned to the non-regular exercise group.

#### Psychological conditions

The Psychosocial Well-Being Index (PWI), which was modified from the General Health Questionnaire score for Koreans, was used to evaluate respondents’ mental health and it has been validated in a large community sample in Korea (Cronbach’s α = .92) [22]. The PWI questionnaire is an 18-item scale designed to evaluate socio-psychological distress symptoms experienced using a four-point response scale (score of 0 = none of the time; score of 3 = all the time). Scores range from 0 to 54, with higher scores indicating higher distress symptoms. Generally, stress levels are categorized into three groups: positive well-being group (PWI ≤ 8), moderate distress group (PWI 9–26), and severe distress group (PWI ≥ 27). Stress events were addressed with the following question: “Have you ever felt stressed that was physically or mentally unbearable during the past month?” Response categories included, not at all, often, and a lot of the time. Self-reported health status was addressed with the following question: “Which statement corresponds to your current health status?” The five response choices were summarized into three categories: healthy (very healthy and healthy), normal, and unhealthy (unhealthy and very unhealthy).

#### Anthropometry

Anthropometric data on height and weight was used to calculate BMI as the Quetelet’s index (kg/m^2^). BMI was split into sex-specific quartiles as follows: Q1 ≤ 22.6, 22.6 < Q2 ≤ 24.4, 24.4 < Q3 ≤ 26.1, and 26.1 < Q4 in men; and Q1 ≤ 21.8, 21.8 < Q2 ≤ 23.5, 23.5 < Q3 ≤ 25.5, 25.5 < Q4 in women. In the same vein, WC (cm) was also classified into quartile groups according to sex distribution: Q1 ≤ 81.3, 81.3 < Q2 ≤ 86.0, 86.0 < Q3 ≤ 91.0, and 91.0 < Q4 in men; and Q1 ≤ 73.5, 73.5 < Q2 ≤ 79.0, 79.0 < Q3 ≤ 84.3, 84.3 < Q4 in women.

#### Health conditions

The HEXA questionnaire covered personal medical history and current medication use for diverse diseases. In the present study, a total of 21 diseases (*i*.*e*., pulmonary tuberculosis, acute liver disease, chronic liver disease, cancer, diabetes mellitus, thyroid disease, hyperlipidemia, depression, cataracts, hypertension, myocardial infarction, stroke, asthma/chronic bronchitis, cholelithiasis, fatty liver disease, gastritis, intestinal polyps, peptic ulcers, arthritis, osteoporosis, and bladder infection) were evaluated. Each disease status was defined by responses to two separate questions: “Have you ever been diagnosed one of the following diseases by a doctor in a hospital?” and “Are you currently undergoing any treatment for the disease?” Current health conditions were summarized into currently getting treatment *vs*. no treatment (*i*.*e*., never diagnosed, completely cured, and receiving no more medication).

### Statistical analysis

To ensure sex-specific differences across the whole analytic study population, baseline characteristics regarding sociodemographic factors, lifestyle, psychological conditions, anthropometry results, and health conditions were first compared according to sex strata. After confirming the statistical significance of sex differences, all analyses were stratified by sex to determine sex-specific effects on the association between putative correlates and sleep duration (S1 and S2 Tables). Selected characteristics for the five domains across sleep duration (<6 hours, 6–7 hours, 8–9 hours, and ≥10 hours) were compared by using chi-square test for categorical variables and analysis of variance (ANOVA) for continuous variables. All results yielding a *p*-value less than 0.05 were considered statistically significant.

Multinomial logistic regression analyses were used to identify the correlates that were significantly associated with short and/or long sleep duration. Along with 6–7 hours of sleep as the comparison group, odds ratios (ORs) and 95% confidence intervals (CIs) were calculated. In all multinomial analyses, the associations with sleep duration were adjusted for all other putative correlates in four domains including sociodemographic factors, lifestyle factors, psychological conditions, and anthropometry results. Correlates included in the fully adjusted models were as follows: age (‘40–49’, ‘50–59’, and ‘60–69’ years), educational attainment (‘college degree or higher’, ‘high school graduate’, and ‘middle school or below’), occupational classification (‘non-manual’, ‘manual’, and ‘unemployed or housewives’), marital status (‘married’ and ‘single’), menopausal status (‘premenopausal’ and ‘postmenopausal’), smoking status (‘non-current smokers’ and ‘current smokers’), alcohol consumption (‘non-current drinkers’ and ‘current drinkers’), eating habits (‘having three meals a day’ and ‘having irregular meals’), multi vitamin intake (‘multi-vitamin users’ and ‘non-users’), physical activity (‘regular exercisers’ and ‘non-exercisers’), PWI status (‘positive wellbeing’, ‘moderate distress’, and ‘severe distress’), stress events (‘not at all’, ‘often’, and ‘frequent’), self-reported health status (‘healthy’, ‘normal’, and ‘unhealthy’), BMI (quartile groups by sex strata), and WC (quartile groups by sex strata). To determine whether the variables were inter-correlated in the models, variance inflation factors (VIFs) for multicollinearity were also computed. After confirming that no multicollinearity was detected (VIFs < 2.0; data not shown), multinomial logistic regression analyses were carried out and nominal *p*-values were estimated. Additionally, considering the multiple hypothesis testing in the present study, the False Discovery Rate (FDR) controlling method was used to adjust spurious association with false positive results. Based on the Benjamini—Hochberg’s method, *p*-values were corrected for multiple comparisons.

Finally, sensitivity analyses were conducted to ensure robustness of the results. Using the three-phase approach to rule out the residual effects of medication use on usual sleep duration, we conducted sensitivity analyses as follows: 1) subjects who were currently taking medication for the diseases that had exhibited a significant association with sleep duration in a univariate analysis, regardless of sex, were excluded from the primary analysis; 2) considering sex-specific associations between medication use and sleep duration, diseases were differently selected by sex strata in univariate analyses and subjects on the medications were excluded accordingly; and 3) subjects who were currently taking any medications for 21 selected diseases above mentioned were wholly excluded from the primary analysis.

All statistical analyses were performed using SAS software version 9.3 (SAS Institute, Cary, North Carolina).

## Results

Baseline characteristics regarding sleep duration, sociodemographic factors, lifestyle, psychological conditions, anthropometry results, and disease-related treatment status are summarized in S1 and S2 Tables. Approximately 65% of men and 60% of women reported a normal sleep duration (6–7 hours); but short or long sleep durations appeared to be fairly common; among men, the proportion of short (<6 hours) and long sleepers (≥10 hours) were 10.4% and 1.9%, respectively; among women, there was a relatively greater proportion of short and long sleepers (12.9% and 2.0%, respectively); the mean age of men was 53.9 years and for women 52.4 years; higher proportions of subjects with a college degree or higher, non-manual occupation, current smokers, current drinkers, and regular exercisers were observed among men (*p* <0.001); while women appeared to have poorer psychological conditions compared to men (*p* <0.001, S1 Table). Prevalence of currently receiving treatment of chronic liver diseases, diabetes mellitus, hypertension, and fatty liver diseases was significantly higher among men; while those of thyroid diseases, depression, and arthritis were significantly higher among women (*p* <0.001, S2 Table).

Distributions of the selected correlates across categories of sleep duration are presented in Tables 1 and 2. Age was inversely correlated with sleep duration for both sexes. Higher proportions of subjects with the lowest education attainment, severe distress, frequent stress events, and self-reported unhealthy status were observed in short and long sleep duration regardless of sex. Current smokers were more frequent in both men and women who slept more than 10 hours per day (Table 1). Subjects who were currently taking medications for diabetes mellitus, depression, hypertension, stroke, peptic ulcer, and arthritis exhibited a significant association with sleep duration regardless of sex. Treatments for cancer and thyroid disease were significantly associated with sleep duration among men; for women, significant associations with sleep duration were observed in medication use on hyperlipidemia, cataract, myocardial infarction, asthma/chronic bronchitis, fatty liver disease, gastritis, osteoporosis, and bladder infection (Table 2).

**Table 1:** Basic characteristics of the study population across categories of sleep duration.

	Men (N = 27,717)					Women (N = 56,377)				
	< 6 hours (N = 2,868)	6–7 hours (N = 17,885)	8–9 hours (N = 6,430)	≥ 10 hours (N = 534)	*P* ^a^	< 6 hours (N = 7,260)	6–7 hours (N = 33,950)	8–9 hours (N = 14,009)	≥ 10 hours (N = 1,158)	*P* ^a^
***Sociodemographic factors***										
**Age (years, mean±SD)**	55.3±8.4	53.3±8.1	54.5±8.0	57.1±7.9	< 0.001	55.0±7.7	52.2±7.5	51.6±7.6	51.7±7.6	< 0.001
**Education attainment, *n* (%)**										
College degree or higher	766 (26.7)	6,233 (34.9)	1,701 (26.5)	73 (13.7)	< 0.001	794 (10.9)	5,542 (16.3)	2,055 (14.6)	108 (9.3)	< 0.001
High school graduate	1,117 (39.0)	7,178 (40.1)	2,657 (41.3)	206 (38.6)		2,396 (33.0)	13,919 (41.0)	5,834 (41.7)	461 (39.8)	
Middle school or below	921 (32.1)	4,137 (23.1)	1,985 (30.9)	248 (46.4)		3,897 (53.7)	13,891 (40.9)	5,834 (41.7)	573 (49.5)	
**Occupational classification, *n* (%)**										
Non-manual	724 (25.2)	5,959 (33.3)	1,591 (24.7)	61 (11.4)	< 0.001	595 (8.2)	3,941 (11.6)	1,087 (7.8)	51 (4.4)	< 0.001
Manual	1,467 (51.2)	8,274 (46.3)	3,115 (48.5)	279 (52.3)		2,050 (28.2)	8,777 (25.9)	2,997 (21.4)	203 (17.5)	
Unemployed / housewives	594 (20.7)	3,199 (17.9)	1,471 (22.9)	179 (33.5)		4,429 (61.0)	20,229 (59.6)	9,394 (67.1)	854 (73.8)	
**Married, *n* (%)**	2,637 (92.0)	16,834 (94.1)	6,086 (94.7)	495 (92.7)	< 0.001	5,725 (78.9)	29,032 (85.5)	12,137 (86.6)	975 (84.2)	< 0.001
**Postmenopausal, *n* (%)**	-	-	-	-	N.A.	4,937 (68.0)	19,066 (56.2)	7,292 (52.1)	586 (50.6)	< 0.001
***Lifestyle factors***										
**Current smokers, n (%)**	864 (30.1)	5,511 (30.8)	2,055 (32.0)	192 (36.0)	0.039	194 (2.7)	669 (2.0)	315 (2.3)	41 (3.5)	< 0.001
**Current drinkers, *n* (%)**	1,957 (68.2)	12,883 (72.0)	4,521 (70.3)	370 (69.3)	0.001	2,077 (28.6)	10,124 (29.8)	4,279 (30.6)	344 (29.7)	0.077
**Having three meals a day, *n* (%)**	2,391 (83.4)	15,702 (87.8)	5,650 (87.9)	440 (82.4)	< 0.001	5,314 (73.2)	27,127 (79.9)	11,132 (79.5)	822 (71.0)	< 0.001
**Multi-vitamin users, *n* (%)**	475 (16.6)	3,099 (17.3)	1,072 (16.7)	54 (10.1)	< 0.001	1,360 (18.7)	7,123 (20.9)	2,647 (18.9)	211 (18.2)	< 0.001
**Regular exercisers, *n* (%)**	1,487 (51.9)	10,172 (56.9)	3,489 (54.3)	224 (42.0)	< 0.001	3,413 (47.0)	17,439 (51.4)	7,117 (50.8)	532 (45.9)	< 0.001
***Psychological condition***										
**PWI status** ^b^ **, *n* (%)**										
Positive wellbeing	380 (13.3)	2,465 (13.8)	946 (14.7)	71 (13.3)	< 0.001	589 (8.1)	3,194 (9.4)	1,422 (10.2)	95 (8.2)	< 0.001
Moderate distress	1,919 (66.9)	13,087 (73.2)	4,517 (70.3)	337 (63.1)		4,648 (64.0)	24,054 (70.9)	9,750 (69.6)	692 (59.8)	
Severe distress	426 (14.9)	1,567 (8.8)	653 (10.2)	96 (18.0)		1,602 (22.1)	4,834 (14.2)	2,052 (14.7)	289 (25.0)	
**Stress events, *n* (%)**										
Not at all	1,458 (50.8)	10,603 (59.3)	3,861 (60.1)	293 (54.9)	< 0.001	2,755 (38.0)	15,897 (46.8)	6,975 (49.9)	523 (45.2)	< 0.001
Often	1,013 (35.3)	5,897 (33.0)	1,982 (30.8)	170 (31.8)		3,065 (42.2)	13,914 (41.0)	5,320 (38.0)	443 (38.3)	
Frequent	322 (11.2)	968 (5.4)	380 (5.9)	53 (9.9)		1,276 (17.6)	3,314 (9.8)	1,303 (9.3)	147 (12.7)	
**Self-reported health status, *n* (%)**										
Healthy	1,176 (41.0)	8,020 (44.8)	2,757 (42.9)	195 (36.5)	< 0.001	1,938 (26.7)	11,180 (32.9)	4,559 (32.5)	316 (27.3)	< 0.001
Normal	1,082 (37.7)	7,432 (41.6)	2,646 (41.2)	196 (36.7)		3,038 (41.9)	15,888 (46.8)	6,273 (44.8)	478 (41.3)	
Unhealthy	573 (20.0)	2,181 (12.2)	920 (14.3)	140 (26.2)		2,158 (29.7)	6,327 (18.6)	2,923 (20.9)	346 (29.9)	
***Anthropometry***										
**BMI (kg/m** ^**2**^ **, mean±SD)**	24.5±2.9	24.4±2.7	24.3±2.7	24.5±2.6	0.003	24.1±3.0	23.7±2.9	23.8±2.9	24.1±3.1	< 0.001
**WC (cm, mean±SD)**	86.5±7.9	86.1±7.3	86.3±7.5	87.6±7.5	< 0.001	80.2±8.5	78.9±8.0	79.2±8.1	80.2±8.4	< 0.001

*Note*: N.A., not applicable; PWI, psychosocial well-being index;BMI, body mass index; WC, waist circumference;

^a^Calculated by chi-square test for categorical variables or analysis of variance (ANOVA) for continuous variables

^b^Defined as Positive wellbeing group: PWI score ≤ 8, Moderate distress group: PWI score 8–27, and Severe distress group: PWI score ≥ 27

**Table 2:** Prevalence of study population currently receiving treatment of diseases across categories of sleep duration.

	Men (N = 27,717)					Women (N = 56,377)				
	< 6 hours (N = 2,868)	6–7 hours (N = 17,885)	8–9 hours (N = 6,430)	≥ 10 hours (N = 534)	*P* ^a^	< 6 hours (N = 7,260)	6–7 hours (N = 33,950)	8–9 hours (N = 14,009)	≥ 10 hours (N = 1,158)	*P* ^a^
***Certain infectious & parasitic disease***										
**Tuberculosis, *n* (%)**	6 (0.2)	10 (0.1)	7 (0.1)	0 (0.0)	0.081	5 (0.1)	15 (0.1)	7 (0.1)	1 (0.1)	0.722
***Viral infections-skin & mucous membrane lesions***										
**Acute liver disease, *n* (%)**	7 (0.2)	26 (0.1)	8 (0.1)	3 (0.6)	0.107	4 (0.1)	11 (0.0)	13 (0.1)	0 (0.0)	0.063
**Chronic liver disease, *n* (%)**	36 (1.3)	154 (0.9)	77 (1.2)	6 (1.1)	0.085	24 (0.3)	143 (0.4)	68 (0.5)	7 (0.6)	0.355
***Neoplasms***										
**Cancer, *n* (%)**	12 (0.4)	82 (0.5)	40 (0.6)	6 (1.1)	0.017	49 (0.7)	236 (0.7)	116 (0.8)	8 (0.7)	0.775
***Endocrine*, *nutritional & metabolic disease***										
**Diabetes mellitus, *n* (%)**	222 (7.7)	1,187 (6.6)	492 (7.7)	65 (12.2)	< 0.001	400 (5.5)	1,325 (3.9)	606 (4.3)	71 (6.1)	< 0.001
**Thyroid disease, *n* (%)**	6 (0.2)	91 (0.5)	40 (0.6)	6 (1.1)	0.048	168 (2.3)	705 (2.1)	331 (2.4)	24 (2.1)	0.419
**Hyperlipidemia, *n* (%)**	78 (2.7)	477 (2.7)	161 (2.5)	21 (3.9)	0.583	253 (3.5)	912 (2.7)	368 (2.6)	27 (2.3)	0.002
***Mental & behavioral disorders***										
**Depression, *n* (%)**	22 (0.8)	50 (0.3)	33 (0.5)	3 (0.6)	0.002	101 (1.4)	215 (0.6)	115 (0.8)	30 (2.6)	< 0.001
***Diseases of the eye & adnexa***										
**Cataract, *n* (%)**	24 (0.8)	110 (0.6)	49 (0.8)	2 (0.4)	0.602	74 (1.0)	214 (0.6)	77 (0.5)	10 (0.9)	< 0.001
***Diseases of the circulatory system***										
**Hypertension, *n* (%)**	612 (21.3)	3,153 (17.6)	1,156 (18.0)	112 (21.0)	< 0.001	1,417 (19.5)	5,044 (14.9)	2,030 (14.5)	194 (16.8)	< 0.001
**Myocardial infarction, *n* (%)**	66 (2.3)	335 (1.9)	145 (2.3)	18 (3.4)	0.051	125 (1.7)	318 (0.9)	123 (0.9)	8 (0.7)	< 0.001
**Stroke, *n* (%)**	39 (1.4)	140 (0.8)	79 (1.2)	15 (2.8)	< 0.001	40 (0.6)	146 (0.4)	72 (0.6)	10 (0.9)	0.048
***Diseases of the respiratory system***										
**Asthma / Chronic bronchitis, *n* (%)**	16 (0.6)	92 (0.5)	45 (0.7)	8 (1.5)	0.088	53 (0.7)	181 (0.5)	77 (0.5)	7 (0.6)	< 0.001
***Diseases of the digestive system***										
**Cholelithiasis, *n* (%)**	4 (0.1)	17 (0.1)	12 (0.2)	1 (0.2)	0.346	8 (0.1)	41 (0.1)	11 (0.1)	3 (0.3)	0.417
**Fatty liver disease, *n* (%)**	31 (1.1)	188 (1.1)	75 (1.2)	8 (1.5)	0.678	41 (0.6)	126 (0.4)	46 (0.3)	9 (0.8)	0.034
**Gastritis, *n* (%)**	54 (1.9)	243 (1.4)	108 (1.7)	11 (2.1)	0.232	188 (2.6)	598 (1.8)	243 (1.7)	21 (1.8)	< 0.001
**Intestinal polyp, *n* (%)**	4 (0.1)	20 (0.1)	9 (0.1)	0 (0.0)	0.420	4 (0.1)	20 (0.1)	7 (0.1)	0 (0.0)	0.895
**Peptic Ulcer, *n* (%)**	32 (1.1)	159 (0.9)	89 (1.4)	5 (0.9)	0.044	83 (1.1)	250 (0.7)	123 (0.9)	8 (0.7)	0.025
***Diseases of the musculo-skeletal system***										
**Arthritis, *n* (%)**	47 (1.6)	198 (1.1)	87 (1.4)	18 (3.4)	< 0.001	494 (6.8)	1,599 (4.7)	699 (5.0)	79 (6.8)	< 0.001
**Osteoporosis, *n* (%)**	11 (0.4)	24 (0.1)	9 (0.1)	1 (0.2)	0.092	292 (4.0)	934 (2.8)	399 (2.8)	42 (3.6)	< 0.001
***Diseases of the genitoruinary system***										
**Bladder infection, *n* (%)**	3 (0.1)	17 (0.1)	13 (0.2)	0 (0.0)	0.494	40 (0.6)	123 (0.4)	54 (0.4)	8 (0.7)	0.041

^a^Chi-square test for categorical variables

After considering the putative effects of habitual characteristics, several correlates were selected as having a significant, direct association with both short and long sleep duration among men (represented by ORs): being older (OR = 1.74, 95% CI = 1.55–1.95 and OR = 2.07, 95% CI = 1.58–2.72, respectively), having the lowest education attainment (OR = 1.38, 95% CI = 1.22–1.56 and OR = 2.74, 95% CI = 2.04–3.68, respectively), having a manual job (OR = 1.15, 95% CI = 1.03–1.28 and OR = 1.76, 95% CI = 1.30–2.37, respectively), having irregular meals (OR = 1.41, 95% CI = 1.26–1.58 and OR = 1.57, 95% CI = 1.24–2.00, respectively), being a non-exerciser (OR = 1.09, 95% CI = 1.00–1.18 and OR = 1.48, 95% CI = 1.23–1.78, respectively), and having poor self-reported health (OR = 1.28, 95% CI = 1.12–1.45 and OR = 1.61, 95% CI = 1.24–2.09, respectively). Among women, both short and long sleep duration were associated with having the lowest education attainment (OR = 1.34, 95% CI = 1.21–1.48 and OR = 1.99, 95% CI = 1.57–2.50, respectively), having irregular meals (OR = 1.50, 95% CI = 1.41–1.59 and OR = 1.59, 95% CI = 1.39–1.82, respectively), severe distress based on PWI status (OR = 1.18, 95% CI = 1.05–1.32 and OR = 1.40, 95% CI = 1.08–1.83, respectively), and poor self-rated health (OR = 1.38, 95% CI = 1.28–1.50 and OR = 1.46, 95% CI = 1.22–1.74, respectively). Interestingly, compared to middle-aged women, elderly women showed a 1.81-fold increase in the odds for short sleep (95% CI = 1.64–2.00) but approximately 40% decrease in the odds for long sleep duration (OR = 0.61, 95% CI = 0.50–0.77; Table 3).

**Table 3:** Odd ratios (95% CIs) ^a^ for short or long sleep duration compared to normal sleep duration (6–7 hours) according to selected correlates.

	Men (N = 27,717)					Women (N = 56,377)				
	< 6 hours	8–9 hours	≥ 10 hours	*P *nominal ^b^	*P *corrected ^c^ ^`^	< 6 hours	8–9 hours	≥ 10 hours	*P *nominal ^b^	*P *corrected ^c^ ^`^
	OR (95% CI)	OR (95% CI)	OR (95% CI)			OR (95% CI)	OR (95% CI)	OR (95% CI)		
***Sociodemographic factors***										
**Age (years)**										
40–49	1.00 (Ref.)	1.00 (Ref.)	1.00 (Ref.)	< 0.001	< 0.001	1.00 (Ref.)	1.00 (Ref.)	1.00 (Ref.)	< 0.001	< 0.001
50–59	1.09 (0.99–1.21)	1.11 (1.03–1.19)	1.56 (1.21–2.00)			1.22 (1.12–1.32)	0.80 (0.76–0.85)	0.70 (0.59–0.83)		
60–69	1.74 (1.55–1.95)	1.10 (1.01–1.20)	2.07 (1.58–2.72)			1.81 (1.64–2.00)	0.76 (0.70–0.82)	0.61 (0.50–0.77)		
**Educational attainment**										
College degree or higher	1.00 (Ref.)	1.00 (Ref.)	1.00 (Ref.)	< 0.001	< 0.001	1.00 (Ref.)	1.00 (Ref.)	1.00 (Ref.)	< 0.001	< 0.001
High school graduate	1.15 (1.03–1.28)	1.27 (1.18–1.37)	1.81 (1.36–2.39)			1.08 (0.99–1.19)	1.05 (0.99–1.12)	1.50 (1.20–1.87)		
Middle school or below	1.38 (1.22–1.56)	1.55 (1.42–1.69)	2.74 (2.04–3.68)			1.34 (1.21–1.48)	1.13 (1.06–1.21)	1.99 (1.57–2.50)		
**Occupational classification**										
Non-manual	1.00 (Ref.)	1.00 (Ref.)	1.00 (Ref.)	< 0.001	< 0.001	1.00 (Ref.)	1.00 (Ref.)	1.00 (Ref.)	< 0.001	< 0.001
Manual	1.15 (1.03–1.28)	1.15 (1.07–1.25)	1.76 (1.30–2.37)			1.10 (0.99–1.23)	1.22 (1.12–1.32)	1.32 (0.96–1.82)		
Unemployed / housewives	1.01 (0.88–1.15)	1.45 (1.32–1.59)	2.77 (2.01–3.83)			1.02 (0.92–1.13)	1.76 (1.63–1.90)	2.88 (2.14–3.88)		
**Marital status**										
Married	1.00 (Ref.)	1.00 (Ref.)	1.00 (Ref.)	< 0.001	0.001	1.00 (Ref.)	1.00 (Ref.)	1.00 (Ref.)	< 0.001	< 0.001
Single or separated	1.26 (1.07–1.48)	0.82 (0.71–0.94)	0.96 (0.67–1.37)			1.19 (1.11–1.27)	0.97 (0.91–1.03)	1.08 (0.91–1.28)		
**Menopausal status**										
Premenopausal	-	-	-	-	-	1.00 (Ref.)	1.00 (Ref.)	1.00 (Ref.)	< 0.001	< 0.001
Postmenopausal	-	-	-			1.14 (1.06–1.24)	0.90 (0.85–0.95)	0.79 (0.67–0.94)		
***Lifestyle factors***										
**Smoking status**										
Non-current smokers	1.00 (Ref.)	1.00 (Ref.)	1.00 (Ref.)	0.017	0.050	1.00 (Ref.)	1.00 (Ref.)	1.00 (Ref.)	0.008	0.019
Current smokers	0.95 (0.87–1.04)	1.07 (1.01–1.15)	1.22 (1.01–1.48)			1.07 (0.90–1.26)	1.13 (0.98–1.30)	1.40 (1.00–1.94)		
**Alcohol consumption**										
Non-current drinkers	1.00 (Ref.)	1.00 (Ref.)	1.00 (Ref.)	0.554	0.764	1.00 (Ref.)	1.00 (Ref.)	1.00 (Ref.)	0.452	0.625
Current drinkers	0.92 (0.85–1.01)	0.96 (0.90–1.02)	1.02 (0.84–1.24)			1.02 (0.96–1.08)	1.02 (0.98–1.07)	0.94 (0.82–1.07)		
**Eating habits**										
Having three meals a day	1.00 (Ref.)	1.00 (Ref.)	1.00 (Ref.)	< 0.001	< 0.001	1.00 (Ref.)	1.00 (Ref.)	1.00 (Ref.)	< 0.001	< 0.001
Having irregular meals	1.41 (1.26–1.58)	1.02 (0.93–1.12)	1.57 (1.24–2.00)			1.50 (1.41–1.59)	1.03 (0.98–1.09)	1.59 (1.39–1.82)		
**Intake of multi vitamin**										
Multi-vitamin users	1.00 (Ref.)	1.00 (Ref.)	1.00 (Ref.)	0.074	0.199	1.00 (Ref.)	1.00 (Ref.)	1.00 (Ref.)	0.004	0.012
Non-users	1.00 (0.90–1.11)	0.97 (0.90–1.05)	1.53 (1.14–2.04)			1.09 (1.02–1.16)	1.09 (1.04–1.15)	1.04 (0.89–1.21)		
**Physical activity**										
Regular exercisers	1.00 (Ref.)	1.00 (Ref.)	1.00 (Ref.)	0.002	0.005	1.00 (Ref.)	1.00 (Ref.)	1.00 (Ref.)	< 0.001	0.002
Non-exercisers	1.09 (1.00–1.18)	1.06 (1.00–1.12)	1.48 (1.23–1.78)			1.04 (0.98–1.10)	1.07 (1.03–1.12)	1.26 (1.11–1.42)		
***Psychological condition***										
**PWI status** ^d^										
Positive wellbeing	1.00 (Ref.)	1.00 (Ref.)	1.00 (Ref.)	< 0.001	< 0.001	1.00 (Ref.)	1.00 (Ref.)	1.00 (Ref.)	< 0.001	< 0.001
Moderate distress	0.92 (0.81–1.04)	0.90 (0.82–0.98)	0.85 (0.65–1.12)			0.96 (0.87–1.05)	0.89 (0.83–0.95)	0.88 (0.70–1.10)		
Severe distress	1.24 (1.04–1.48)	1.00 (0.87–1.14)	1.30 (0.90–1.87)			1.18 (1.05–1.32)	0.88 (0.80–0.96)	1.40 (1.08–1.83)		
**Stress events**										
Not at all	1.00 (Ref.)	1.00 (Ref.)	1.00 (Ref.)	< 0.001	< 0.001	1.00 (Ref.)	1.00 (Ref.)	1.00 (Ref.)	< 0.001	< 0.001
Often	1.21 (1.10–1.32)	0.92 (0.86–0.98)	0.96 (0.78–1.17)			1.14 (1.08–1.21)	0.88 (0.84–0.92)	0.85 (0.75–0.98)		
Frequent	1.84 (1.58–2.13)	0.96 (0.84–1.09)	1.13 (0.81–1.57)			1.59 (1.46–1.73)	0.87 (0.81–0.94)	0.91 (0.74–1.11)		
**Self-reported health status**										
Healthy	1.00 (Ref.)	1.00 (Ref.)	1.00 (Ref.)	< 0.001	< 0.001	1.00 (Ref.)	1.00 (Ref.)	1.00 (Ref.)	< 0.001	< 0.001
Normal	0.92 (0.84–1.01)	1.01 (0.95–1.08)	0.95 (0.77–1.17)			1.02 (0.95–1.09)	0.98 (0.94–1.03)	0.96 (0.83–1.12)		
Unhealthy	1.28 (1.12–1.45)	1.10 (1.00–1.22)	1.61 (1.24–2.09)			1.38 (1.28–1.50)	1.18 (1.11–1.26)	1.46 (1.22–1.74)		
***Anthropometry***										
**Body mass index** ^e^ **(kg/m2)**										
Quartile 1 (Lowest)	1.00 (0.88–1.14)	0.99 (0.91–1.08)	0.71 (0.54–0.95)	< 0.001	< 0.001	0.99 (0.91–1.08)	1.04 (0.98–1.10)	0.92 (0.76–1.11)	0.282	0.461
Quartile 2	1.00 (Ref.)	1.00 (Ref.)	1.00 (Ref.)			1.00 (Ref.)	1.00 (Ref.)	1.00 (Ref.)		
Quartile 3	1.07 (0.94–1.21)	0.94 (0.86–1.02)	0.88 (0.68–1.13)			1.01 (0.93–1.09)	1.03 (0.97–1.09)	0.93 (0.78–1.11)		
Quartile 4 (Highest)	1.32 (1.15–1.51)	0.87 (0.79–0.96)	0.67 (0.50–0.90)			1.09 (1.00–1.19)	1.01 (0.94–1.08)	1.00 (0.81–1.22)		
**Waist circumference** ^f^ **(cm)**										
Quartile 1 (Lowest)	1.03 (0.90–1.17)	0.92 (0.84–1.01)	0.95 (0.71–1.27)	< 0.001	0.002	1.06 (0.98–1.15)	0.95 (0.89–1.01)	0.91 (0.76–1.10)	0.109	0.231
Quartile 2	1.00 (Ref.)	1.00 (Ref.)	1.00 (Ref.)			1.00 (Ref.)	1.00 (Ref.)	1.00 (Ref.)		
Quartile 3	0.95 (0.85–1.08)	0.96 (0.88–1.04)	1.11 (0.85–1.44)			1.03 (0.95–1.11)	1.00 (0.94–1.06)	1.03 (0.86–1.23)		
Quartile 4 (Highest)	0.96 (0.84–1.11)	1.09 (0.98–1.20)	1.66 (1.23–2.23)			1.00 (0.92–1.10)	1.07 (1.00–1.15)	1.21 (0.98–1.48)		

*Note*: Unknown values were wholly included in the statistical models but were not presented in the table

^a^All ORs were adjusted for sociodemographic factors (*i*.*e*., age, education attainment, occupational classification, marital status, and menopausal status for women only), lifestyle factors (*i*.*e*., smoking status, alcohol consumption, eating habits, intake of multi vitamin, and physical activity), psychological conditions (*i*.*e*., PWI status, stress events, and self-reported health status), and anthropometry (*i*.*e*., body mass index and waist circumference)

^b^Estimated in multinomial logistic regression models according to a nominal scale

^c^Computed by using the Benjamini—Hochberg’s false discovery rate (FDR) controlling method

^d^Defined as Positive wellbeing group: PWI score ≤ 8, Moderate distress group: PWI score 8–27, and Severe distress group: PWI score ≥ 27

^e^Defined as Quartile 1: BMI ≤ 22.6 kg/cm^2^, Quartile 2: 22.6 kg/cm^2^ < BMI ≤ 24.4 kg/cm^2^, Quartile 3: 24.4 kg/cm^2^ < BMI ≤ 26.1 kg/cm^2^, and Quartile 4: BMI > 26.1 kg/cm^2^ in men; Quartile 1: BMI ≤ 21.8 kg/cm^2^, Quartile 2: 21.8 kg/cm^2^ < BMI ≤ 23.5 kg/cm^2^, Quartile 3: 23.5 kg/cm^2^ < BMI ≤ 25.5 kg/cm^2^, and Quartile 4: BMI >25.5 kg/cm^2^ in women

^f^Defined as Quartile 1: WC ≤ 81.3cm, Quartile 2: 81.3cm < WC ≤ 86.0cm, Quartile 3: 86.0cm < WC ≤ 91.0cm, and Quartile 4: WC > 91.0cm in men; Quartile 1: WC ≤ 73.5cm, Quartile 2: 73.5cm < WC ≤ 79.0cm, Quartile 3: 79.0cm < WC ≤ 84.3cm, Quartile 4: 84.3cm < WC in women

Single correlates for short or long sleep duration among men were as follows: being single (OR = 1.26, 95% CI = 1.07–1.48), having severe distress based on PWI status (OR = 1.24, 95% CI = 1.04–1.48), having frequent stress events (OR = 1.84, 95% CI = 1.58–2.13) and being in the highest BMI quartile group (OR = 1.32, 95% CI = 1.15–1.51) raised the odds of having short sleep duration. In contrast, being unemployed (OR = 2.77, 95% CI = 2.01–3.83), being a current smoker (OR = 1.22, 95% CI = 1.01–1.48), consuming multi-vitamins (OR = 1.53, 95% CI = 1.14–2.04), and being in the highest viscerally obese group (OR = 1.66, 95% CI = 1.23–2.23) raised the odds of having long sleep duration. Most of the selected correlates for short or long sleep duration among men were reproduced in women: being single (OR = 1.19, 95% CI = 1.11–1.27), frequent stress events (OR = 1.59, 95% CI = 1.46–1.73), and being in the highest BMI quartile group (OR = 1.09, 95% CI = 1.00–1.19) raised the odds of having short sleep duration, whereas being unemployed or housewives (OR = 2.88, 95% CI = 2.14–3.88), being a current smoker (OR = 1.40, 95% CI = 1.00–1.94), and not exercising (OR = 1.26, 95% CI = 1.11–1.42) raised the odds of having long sleep duration. Postmenopausal status was inversely correlated with sleep duration (OR = 1.14, 95% CI = 1.06–1.24 for short sleep and OR = 0.79, 95% CI = 0.67–0.94 for long sleep; Table 3).

Those currently receiving treatment for diabetes had significantly increased odds of having long sleep duration, regardless of sex (OR = 1.37, 95% CI = 1.04–1.81 for men and OR = 1.43, 95% CI = 1.11–1.85 for women). Men who were undergoing treatment for thyroid disease were less likely to be short sleepers (OR = 0.39, 95% CI = 0.17–0.90), but men taking medication for depression were more likely to be short sleepers (OR = 1.69, 95% CI = 1.01–2.85). Treatment for depression appeared to be associated with increased odds of having both short and long sleep duration among women (OR = 1.54, 95% CI = 1.21–1.97 for short sleep and OR = 2.85, 95% CI = 1.92–4.25 for long sleep). Among men, medical treatments for stroke and arthritis was associated with higher odds of having long sleep duration (OR = 1.77, 95% CI = 1.01–3.10 and OR = 1.90, 95% CI = 1.15–3.15; Table 4).

**Table 4:** Odd ratios (95% CIs) ^a^ for short or long sleep duration compared to normal sleep duration (6–7 hours) according to currently receiving treatment of diseases.

	Men (N = 27,717)					Women (N = 56,377)				
	< 6 hours	8–9 hours	≥ 10 hours	*P *nominal ^b^	*P *corrected ^c^	< 6 hours	8–9 hours	≥ 10 hours	*P *nominal ^b^	*P *corrected ^c^
	OR (95% CI)	OR (95% CI)	OR (95% CI)			OR (95% CI)	OR (95% CI)	OR (95% CI)		
**Tuberculosis**	2.25 (0.80–6.34)	1.54 (0.58–4.11)	- ^d^	0.399	0.633	1.49 (0.53–4.18)	1.17 (0.47–2.88)	2.02 (0.26–15.5)	0.824	0.873
**Acute liver disease**	1.28 (0.55–3.01)	0.74 (0.33–1.63)	2.34 (0.67–8.12)	0.417	0.633	1.30 (0.40–4.21)	2.68 (1.19–6.04)	- ^d^	0.147	0.295
**Chronic liver disease**	1.26 (0.87–1.83)	1.30 (0.98–1.72)	0.99 (0.43–2.27)	0.663	0.800	0.69 (0.45–1.08)	1.11 (0.83–1.49)	1.29 (0.60–2.79)	0.333	0.521
**Cancer**	0.70 (0.38–1.30)	1.18 (0.81–1.74)	1.67 (0.71–3.93)	0.132	0.283	0.92 (0.67–1.26)	1.15 (0.92–1.45)	0.86 (0.42–1.75)	0.794	0.866
**Diabetes mellitus**	0.99 (0.84–1.15)	1.05 (0.94–1.18)	1.37 (1.04–1.81)	0.273	0.503	1.03 (0.92–1.16)	1.11 (1.00–1.23)	1.43 (1.11–1.85)	0.003	0.009
**Thyroid disease**	0.39 (0.17–0.90)	1.18 (0.81–1.71)	2.05 (0.88–4.79)	0.138	0.283	1.07 (0.90–1.28)	1.13 (0.99–1.29)	0.93 (0.61–1.40)	0.515	0.657
**Hyperlipidemia**	0.91 (0.71–1.17)	0.93 (0.78–1.12)	1.33 (0.84–2.09)	0.716	0.804	1.04 (0.90–1.20)	1.01 (0.89–1.14)	0.84 (0.57–1.24)	0.608	0.706
**Depression**	1.69 (1.01–2.85)	1.62 (1.03–2.53)	1.00 (0.30–3.29)	0.349	0.583	1.54 (1.21–1.97)	1.26 (1.00–1.58)	2.85 (1.92–4.25)	< 0.001	< 0.001
**Cataract**	1.12 (0.71–1.76)	1.12 (0.80–1.58)	0.44 (0.11–1.80)	0.688	0.803	1.09 (0.83–1.43)	0.90 (0.69–1.17)	1.23 (0.64–2.34)	0.475	0.633
**Hypertension**	1.07 (0.97–1.19)	0.95 (0.88–1.02)	0.88 (0.71–1.11)	0.123	0.283	1.03 (0.96–1.11)	1.00 (0.94–1.06)	1.09 (0.92–1.29)	0.923	0.923
**Myocardial infarction**	0.93 (0.70–1.22)	1.06 (0.87–1.30)	1.11 (0.67–1.82)	0.589	0.764	1.22 (0.98–1.51)	0.94 (0.76–1.16)	0.61 (0.30–1.25)	0.246	0.443
**Stroke**	1.16 (0.81–1.68)	1.28 (0.97–1.70)	1.77 (1.01–3.10)	0.219	0.427	0.83 (0.58–1.18)	1.12 (0.84–1.49)	1.43 (0.75–2.75)	0.368	0.541
**Asthma / Chronic bronchitis**	0.80 (0.47–1.38)	1.19 (0.83–1.71)	1.82 (0.86–3.84)	0.571	0.764	1.05 (0.76–1.43)	1.02 (0.78–1.34)	0.96 (0.45–2.06)	0.007	0.019
**Cholelithiasis**	0.96 (0.32–2.90)	1.66 (0.79–3.52)	1.26 (0.16–9.63)	0.758	0.804	0.76 (0.35–1.64)	0.64 (0.33–1.24)	1.91 (0.59–6.22)	0.655	0.737
**Fatty liver disease**	0.91 (0.62–1.34)	1.03 (0.79–1.36)	1.18 (0.57–2.43)	0.854	0.854	1.15 (0.80–1.65)	0.88 (0.62–1.23)	1.80 (0.90–3.58)	0.584	0.701
**Gastritis**	1.21 (0.90–1.64)	1.14 (0.91–1.44)	1.20 (0.65–2.24)	0.821	0.845	1.13 (0.95–1.34)	0.98 (0.84–1.14)	0.87 (0.56–1.36)	0.267	0.458
**Intestinal polyp**	1.23 (0.41–3.65)	1.24 (0.56–2.75)	- ^d^	0.661	0.800	0.75 (0.25–2.24)	0.81 (0.34–1.92)	- ^d^	0.877	0.902
**Peptic Ulcer**	1.01 (0.68–1.49)	1.43 (1.10–1.86)	0.76 (0.31–1.88)	0.106	0.264	1.15 (0.89–1.49)	1.18 (0.95–1.47)	0.75 (0.37–1.52)	0.529	0.657
**Arthritis**	1.08 (0.78–1.50)	1.05 (0.81–1.35)	1.90 (1.15–3.15)	0.293	0.512	1.02 (0.91–1.13)	1.07 (0.97–1.17)	1.25 (0.98–1.59)	0.033	0.076
**Osteoporosis**	1.99 (0.95–4.15)	0.89 (0.41–1.92)	0.77 (0.10–5.88)	0.559	0.764	1.04 (0.90–1.19)	1.07 (0.95–1.21)	1.24 (0.90–1.72)	0.376	0.541
**Bladder infection**	0.76 (0.22–2.64)	1.81 (0.87–3.76)	- ^d^	0.756	0.804	1.26 (0.87–1.81)	1.06 (0.77–1.46)	1.65 (0.80–3.40)	0.185	0.351

*Note*: Unknown values were wholly included in the statistical models but were not presented in the table

^a^All ORs were adjusted for sociodemographic factors (*i*.*e*., age, education attainment, occupational classification, marital status, and menopausal status for women only), lifestyle factors (*i*.*e*., smoking status, alcohol consumption, eating habits, intake of multi vitamin, and physical activity), psychological condition (*i*.*e*., PWI status, stress events, and self-reported health status), and anthropometry (*i*.*e*., body mass index and waist circumference)

^b^Estimated in multinomial logistic regression models according to a nominal scale

^c^Computed by using the Benjamini—Hochberg’s false discovery rate (FDR) controlling method

^d^Estimate was not provided due to the cell with zero count

After adjusting for multiple comparisons, significant associations between putative correlates and sleep duration were still observed, especially for sociodemographic factors and psychological conditions in both sexes. However, all adjusted *p*-values for health conditions failed to reach the level of statistical significance among men. Among women, current treatments for diabetes mellitus and depression appeared to be correlated with variations in the usual sleep duration when multiple comparisons were controlled (*p* corrected = 0.009 and < 0.001, respectively; Tables 3 and 4).

Three-phase sensitivity analyses indicate that all results are robust to residual effects of medication use and/or comorbidity status on usual sleep duration. Generally comparable trends were achieved regardless of underlying assumptions, though minor variations in each effect size were observed (S3 and S4 Tables).

## Discussion

The present study attempted to identify characteristics within five domains (*i*.*e*., sociodemographic factors, lifestyle factors, psychological conditions, anthropometry, and health conditions) that might be simultaneously and/or independently associated with short or long sleep duration among middle-aged and elderly Koreans. Regardless of sexual differences, adverse behaviors and lifestyle factors including being unmarried, having low socioeconomic status, currently smoking, not exercising, having irregular meals, and having poor psychosocial well-being, experiencing frequent stress events, or having poor self-rated health all raised the odds of having an abnormal sleep duration. Furthermore, diabetes mellitus and depression were positively associated with abnormal sleep duration in both men and women. The present findings have indicated that a substantial proportion of the middle-aged and elderly Korean populations may be affected by deleterious effects of inadequate sleep duration. We found that 12.1% of the study population slept less than 6 hours (10.4% for men and 12.9% for women) and 2.0% slept 10 hours and more (1.9% for men and 2.0% for women). Overall prevalence of short and long sleepers followed a similar trend when compared to the statistics derived from the Fourth Korea National Health and Nutrition Examination Survey (KNHANES IV, 2007–2009); but absolute proportions for short and long sleep duration were higher than those of ours (data not shown). This can be explained by the healthy volunteer effect in the HEXA study: our participants recruited for the health examination centers may be more likely to be concerned with health status and to engage in health-seeking behaviors compared to the general population. In order to encourage good physical health and psychosocial well-being, people who sleep too little or too much should be treated as at-risk populations and a target for tailor-made interventions involving risk modification on sleep.

### Sociodemographic factors

In general, aging is assumed to influence certain variations in sleep duration, quality, and architecture due to physiological age-dependent changes in circadian rhythms, homeostatic regulation, respiratory functioning, and endocrine functioning [23]. Age appears to be a predictor of short and long sleep duration, but the influence of age may differ by sex. Studies have consistently reported that total sleep duration decreases with age [24,25], particularly among women [18]. However, the controversy as to whether aging is significantly linked to long sleep duration has not been resolved; some studies continue to show that long sleepers are more prevalent in elderly men [26,27]. Further studies focusing on the biological mechanisms of sleep structure by age are warranted.

Socioeconomic status is assumed to play a crucial role in sleep duration; having a low socioeconomic status has been shown to correlate with both short and long sleep duration even after adjusting for other physical/psychological health-related characteristics [19,20,28], which is in line with the present findings. Socioeconomic status might help stimulate the motivation for healthy behaviors; additionally, higher socioeconomic status is assumed to provide individuals with health-promoting resources to avoid risky behaviors [29]. In the same vein, higher socioeconomic status would mediate the association between adverse sleep duration and healthy behaviors. This in turn helps avoid inadequate sleep duration. Although we could not clarify the underlying mechanisms linking socioeconomic status to sleep structure, our findings suggest that socioeconomic disparities are detrimental to sleep duration. Interestingly, being unemployed appeared to be associated with a nearly threefold increase in the odds of having longer sleep duration in both sexes. This can be reasonably explained by unemployed individuals being undeterred by a fixed work schedule; thus, these subjects can have the opportunity to sleep more than employed individuals. Consistent with our findings, study in Finland demonstrated that occupation was associated with both short and long sleep duration [30]; being unemployed predicted a 2.42-fold increase in longer sleep duration [31]. Furthermore, being a housewife was associated with both short and long sleep duration among a sample of Chinese women [18].

In terms of marital status, being unmarried was a significant correlate of short sleep duration for both men and women; this is consistent with other studies [3,32]. Marital status was reported to be an independent risk factor for low sleep quality, while those living with a spouse or family member tend to have better sleep quality [33,34]. Marriage is generally regarded as a key social relationship in adulthood, which can be a protective factor against risks of morbidity and/or mortality [35]. Notably, support from other relationships cannot fully compensate for the effect of being single [35]; we suppose that sleep deprivation and disturbance might be greater in single people because they might have overall poorer psychological conditions and/or engage in more unhealthy behaviors.

Postmenopausal women appear to frequently experience a significant decrease in sleep quantity. Generally, women in menopausal transition and in the postmenopausal period are reported to have sleep problems such as shorter duration, low sleep efficiency, overall low quality, and insomnia complaints [36–38]. This phenomenon may be explained by hormonal changes, climacteric symptoms, or ageing effect accompanying changes in circadian rhythms and physiological functions. Studies focused on sleep physiology at different stages of a woman's life cycle will further clarify the complex mechanism.

### Lifestyle factors

In the present study, current smoking was related to long sleep duration for both men and women. The association between smoking and long sleep has been controversial [18,21,39]—in contrast to our findings, current smoking has been reported as a correlate of short sleep duration. Nevertheless, smokers appear more likely to suffer from disturbed sleep, regardless of sex. Studies have consistently indicated that smoking can independently lead to disturbed sleep architecture for both men and women [39,40]. An integrated approach to understand the underlying dynamics between smoking and sleep duration is necessary. Although alcohol consumption was not associated with abnormal sleep duration in the present study, previous studies have demonstrated associations between alcohol consumption and short [41] and long sleep duration [31]. To better clarify the association between alcohol consumption and sleep duration, the quantity of alcohol consumption should be considered in further studies.

Having irregular meals was a meaningful correlate of both short and long sleep duration in the present study. Generally, short sleepers are assumed to engage in more irregular meals and frequent snacking [42]. Long sleep duration has been also reported to correlate with unconventional eating rhythms [17]. Given that sleep irregularity, uncontrolled diet, and psychological stress are interrelated, this cycle may be alleviated by behavioral sleep modification or simple diet modifications. The association between multi-vitamin intake and sleep duration differed by sex; men showed significant associations with long sleep duration while women participants showed associations with short sleep duration. A plausible explanation of these findings is reverse causation: unhealthy individuals with sleep problems might take health supplements (like multi-vitamins) [18]; thus, multi-vitamin intake may be associated with general abnormal sleep duration. Further studies that include detailed information are needed to clarify their association with sleep patterns.

Regular exercise was revealed to be associated with a lower likelihood of abnormal sleep duration. It is generally thought that physical exercise can be a therapy comparable to pharmacological treatments for treating sleep disorders and depression [43,44]. Furthermore, a recent study demonstrated a dose-response relationship between exercise and sleep episodes, whereby higher levels of exercise influence favorable sleep patterns, including higher sleep quality, shortened sleep latency, fewer awakenings, and better psychological functioning [45]. These associations can be explained via several biological mechanisms [43–46]: exercise is assumed to (1) affect endogenous concentration of melatonin, which is related to circadian rhythms; (2) promote sleep-wake homeostatic regulation; (3) play a crucial role in thermoregulation linked to sleep; and (4) increase the secretion of endogenous opioid peptides (β- endorphin) in the brain. Common benefits of physical exercise appear to activate physiological and/or psychological alterations, which can produce positive alterations connected with improved sleep duration.

### Psychological conditions

Poor psychological conditions, such as severe distress as measured by the PWI, frequent stress events, and poor self-rated health, had consistent associations with abnormal sleep duration. These results confirm past results showing that (1) psychosocial stressors could be a key component to changes in sleep architecture (*i*.*e*., increased sleep latency, decreased sleep efficiency, decreased rapid eye movement (REM) and slow-wave sleep, and increased frequency of awakening) that are closely correlated with impaired and disturbed sleep [47–49]; and (2) short and long sleep are positively associated with poor self-rated health, even after adjusting for other confounding factors such as age, gender, socioeconomic status, lifestyle habits, and mental conditions [27,50]. Sleep disturbances also show a significantly inverse association with fair self-rated health [51]. These psychological factors systematically interfere with each other, leading to impaired sleep duration in both a dependent and/or independent manner. Furthermore, given that mental health directly affects physiological health, psychological factors may act as a modifier for typical sleep episodes. Hypothetically, the causal link between psychological distress and sleep problems is assumed to be mediated by emotional stability; diverse supportive relationships can provide emotional comfort and attenuate stress responses in sleep [52,53]. Such factors are also able to buffer against the harms of psychological distress and risky health behaviors [54]. Additionally, health-promoting effects can be induced even with fair sleep conditions. In order to draw a definitive conclusion regarding causal associations, future studies assessing more detailed psychological health information and other confounding factors, including disease comorbidity, quality of life, and social networks, should be conducted with objective measures of sleep structure.

### Health conditions

Although we cannot clarify the causal relationships due to the limits of a cross-sectional study design, our findings revealed that sleep duration could be negatively influenced by the presence of disease and/or drug treatment. In the present study, diabetes mellitus was significantly associated with long sleep duration in both men and women; furthermore, depression was linked to adverse sleep duration regardless of sex. Previous studies have reported that (1) several health conditions, such as hypertension, coronary heart disease, stroke, diabetes, gout, hyperlipidemia, fatty liver, and a history of cancer were significantly associated with short and/or long sleep duration among Chinese women population [18]; (2) short sleep duration was more frequently observed among elderly Taiwanese who had high depression scores while heart disease was related to long sleep duration [29]; and (3) some chronic diseases, including obesity, diabetes, hypertension, and cardiovascular disease, were associated with short and long sleep duration among U.S. adults [55]. The link between sleep and adverse health outcomes can be explained by biological alterations related to inadequate sleep duration: (1) sleep impairments significantly affect the normal functioning of daily metabolic and hormonal processes by changes in circulating levels of various hormones [56]; (2) altered hormones levels are linked to abnormal appetite regulation, impaired glucose metabolism, and disturbances in immune function [56–59]; and (3) cortisol secretion is remarkably increased, which promotes the development of the metabolic and cognitive consequences of glucocorticoid excess [60,61]. Further longitudinal follow-up and biological studies will help evaluate the long-term effects of adverse sleep duration on health.

Several study limitations should be noted. First, because the present study was based on a cross-sectional analysis from a prospective cohort study, we could not determine the causal relationships and chronological variations between sleep duration and its correlates. Second, although a regular intake of pharmaceutical drugs such as sleeping pills and antidepressants is known to affect sleep quantity as well as quality, we could not consider pharmaceutical status in our analyses due to lacking the information. Furthermore, the caffeine effects in sleep disruption [62] could not be explored in the present study due to lack of details on caffeine use including dietary sources of caffeine and the average amount of caffeine consumed in everyday life. Given this limitation, our findings should be interpreted with caution. Third, we collected sleep duration data based on a single question separated into specific categories: <6 hours, 6–7 hours, 8–9 hours, and ≥10 hours. This limited information made it difficult to provide more specific analyses for evaluating the relationships between sleep duration and its correlates, thereby resulting in less accurate results. For example, we did not calculate mean sleep duration nor did we distinguish sleep duration on weekdays from that on weekends. In spite of these limitations however, the cross-sectional analysis of a large-scale, population-based cohort provides adequate power. Hence, we were able to conduct an analysis on sleep duration by assessing a wide range of sociodemographic factors, lifestyles, psychological factors, and various health conditions. Our approach allowed us to reveal significant correlates of sleep duration among the middle-aged and elderly Korean population, which may help extend our understanding of other Asian populations regarding sleep duration. Moreover, considering how individual characteristics appear to be inter-correlated with sociocultural aspects in a complex manner, the study findings could provide clues to estimate the sociocultural factors involved with inadequate sleep duration in a developed, non-Western society.

## Conclusion

Detrimental health behaviors and lifestyle factors, such as being unmarried, having low socioeconomic status, currently smoking, not exercising, having irregular meals, having poor psychosocial conditions, and receiving medical treatment for diabetes and depression, might be meaningful correlates of abnormal sleep duration among middle-aged and elderly Koreans. Further studies assessing factors related to sleep and its proximal and distal consequences should be explored in depth while considering other dimensions (*e*.*g*., sleep structure and architecture) beyond sleep duration.

## Supporting Information

S1 Table Basic characteristics of the study population. (PDF)

S2 Table Prevalence of currently receiving treatment of selected diseases of the study population. (PDF)

S3 Table Phase sensitivity analysis among men: odd ratios (95% CIs) for short or long sleep duration compared to normal sleep duration (6–7 hours) according to selected correlates. (PDF)

S4 Table Phase sensitivity analysis among women: odd ratios (95% CIs) for short or long sleep duration compared to normal sleep duration (6–7 hours) according to selected correlates. (PDF)
