# Supplementary material for: Correlates of Self-Reported Sleep Duration in Middle-Aged and Elderly Koreans: from the Health Examinees Study
Source: PLoS One. 2015 May 1;10(5):e0123510. doi: 10.1371/journal.pone.0123510 (PMC4416918; doi:10.1371/journal.pone.0123510)
Supplement: S1 Table — (PDF) [file pone.0123510.s001.pdf]

S1 Table. Basic characteristics of the study population

	Men (N=27,717)	Women (N=56,377)	*P* [a]
Sleep duration			
**Hours per day**			<0.001
< 6 hours	2,868 (10.4)	7,260 (12.9)	
6-7 hours	17,885 (64.5)	33,950 (60.2)	
8-9 hours	6,430 (23.2)	14,009 (25.9)	
≥ 10 hours	534 (1.9)	1.158 (2.0)	
Sociodemographic factors			
**Age (years, mean±SD)**	53.9 ± 8.2	52.4 ± 7.6	<0.001
40-49	9,372 (33.8)	22,344 (39.6)	
50-59	10,335 (37.3)	22,340 (39.6)	
60-69	8,010 (28.9)	11,693 (20.7)	
**Educational attainment**			<0.001
Middle school or below	7,291 (26.3)	24,195 (42.9)	
High school graduate	11.158 (40.3)	22,610 (40.1)	
College degree or higher	8,773 (31.6)	8,499 (15.1)	
Unknown	495 (1.8)	1,073 (1.9)	
**Occupational classification**			<0.001
Non-manual	8,335 (30.1)	5,674 (10.1)	
Manual	13,135 (47.4)	14,027 (24.9)	
Unemployed / housewives	5,443 (19.6)	34,906 (61.9)	
Unknown	804 (2.9)	1,770 (3.1)	
**Marital status**			<0.001
Married	26,052 (94.0)	47,869 (84.9)	
Single or separated	1,441 (5.2)	8,079 (14.3)	
Unknown	224 (0.8)	429 (0.7)	
Lifestyle factors			
**Smoking status**			<0.001
Non-current smokers	18.997 (68.5)	54,654 (96.9)	
Current smokers	8,622 (31.1)	1,219 (2.2)	
Unknown	98 (0.4)	504 (0.9)	
**Alcohol consumption**			<0.001
Non-current drinkers	7,931 (28.6)	39,205 (69.5)	
Current drinkers	19,731 (71.2)	16,824 (29.8)	
Unknown	55 (0.2)	348 (0.6)	
**Eating habits**			<0.001
Having three meals a day	24,183 (87.3)	44,395 (78.7)	
Having irregular meals	3,457 (12.5)	11,843 (21.0)	
Unknown	77 (0.3)	139 (0.3)	
**Intake of multi vitamin**			<0.001
Multi-vitamin users	4,700 (17.0)	11,341 (20.1)	
Non-users	22,659 (81.7)	44,153 (78.3)	
Unknown	358 (1.3)	883 (1.6)	
**Physical activity**			<0.001
Regular exercisers	15,372 (55.5)	28,501 (50.6)	
Non-exercisers	12,291 (44.3)	27,766 (49.3)	
Unknown	54 (0.2)	110 (0.2)	
Psychological condition			
**PWI status** [b]			<0.001
Positive wellbeing	3,862 (13.9)	5,300 (9.4)	
Moderate distress	19,860 (71.7)	39,144 (69.4)	
Severe distress	2,742 (9.9)	8,777 (15.6)	
Unknown	1,253 (4.5)	3,156 (5.6)	

**Stress events**			<0.001
Not at all	16,215 (58.5)	26,150 (46.4)	
Often	9,062 (32.7)	22,742 (40.3)	
Frequent	1,723 (6.2)	6,040 (10.7)	
Unknown	717 (2.6)	1,445 (2.6)	
**Self-reported health status**			<0.001
Healthy	12,148 (43.8)	17,993 (31.9)	
Normal	11,356 (41.0)	25,677 (45.6)	
Unhealthy	3,814 (13.8)	11,754 (20.9)	
Unknown	399 (1.4)	953 (1.7)	
Anthropometry			
**Body mass index (kg/m^2, mean±SD)** [c]	24.4 ± 2.7	23.8 ± 2.9	<0.001
Quartile 1 (Lowest)	7,000 (25.3)	14,447 (25.6)	
Quartile 2	7,161 (25.8)	13,739 (24.4)	
Quartile 3	6,584 (23.8)	14,062 (24.9)	
Quartile 4 (Highest)	6,737 (24.3)	13,722 (24.3)	
Unknown	235 (0.8)	407 (0.7)	
**Waist circumference (cm, mean±SD)** [d]	86.2 ± 7.4	79.2 ± 8.1	<0.001
Quartile 1 (Lowest)	6,757 (24.4)	13,944 (24.7)	
Quartile 2	6,885 (24.8)	15,246 (27.0)	
Quartile 3	6,893 (24.9)	12,311 (21.8)	
Quartile 4 (Highest)	6,482 (23.4)	13,789 (24.5)	
Unknown	700 (2.5)	1,087 (1.9)	

a. *p*-value was calculated by chi-square test for categorical variables or Student's *t*-test for continuous variables

b. Defined as positive wellbeing group with PWI score ≤ 8, moderate distress group with 8 < PWI score < 27, and severe distress group with PWI score ≥ 27

c. Defined as follows: Quartile 1: ≤ 22.6, Quartile 2: 22.6 < BMI ≤ 24.4, Quartile 3: 24.4 < BMI ≤ 26.1, and Quartile 4: 26.1< BMI in men; and Quartile 1: ≤ 21.8, Quartile 2: 21.8 < BMI ≤ 23.5, Quartile 3: 23.5 < BMI ≤ 25.5, and Quartile 4: 25.5 < BMI in women

d. Defined as follows: Quartile 1: ≤ 81.3, Quartile 2: 81.3 < WC ≤ 86.0, Quartile 3: 86.0 < BMI ≤ 91.0, and Quartile 4: 91.0 < BMI in men; and Quartile 1: ≤ 73.5, Quartile 2: 73.5 < WC ≤ 79.0, Quartile 3: 79.0 < WC ≤ 84.3, Quartile 4: 84.3 < WC in women
