# Supplementary material for: Correlates of Self-Reported Sleep Duration in Middle-Aged and Elderly Koreans: from the Health Examinees Study
Source: PLoS One. 2015 May 1;10(5):e0123510. doi: 10.1371/journal.pone.0123510 (PMC4416918; doi:10.1371/journal.pone.0123510)
Supplement: S2 Table — (PDF) [file pone.0123510.s002.pdf]

S2 Table. Prevalence of currently receiving treatment of selected diseases of the study population

	Men (N=27,717)	Women (N=56,377)	*P* [a]
Certain infectious & parasitic disease			
**Tuberculosis, *n* (%)**	23 (0.1)	28 (0.1)	< 0.001
Viral infections characterized by skin & mucous membrane lesions			
**Acute liver disease, *n* (%)**	44 (0.2)	28 (0.1)	< 0.001
**Chronic liver disease, *n* (%)**	273 (1.0)	242 (0.4)	< 0.001
Neoplasms			
**Cancer, *n* (%)**	140 (0.5)	409 (0.7)	< 0.001
Endocrine, nutritional & metabolic disease			
**Diabetes mellitus, *n* (%)**	1.966 (7.1)	2,402 (4.3)	< 0.001
**Thyroid disease, *n* (%)**	143 (0.5)	1,228 (2.2)	< 0.001
**Hyperlipidemia, *n* (%)**	737 (2.7)	1,560 (2.7)	0.130
Mental & behavioral disorders			
**Depression, *n* (%)**	108 (0.4)	461 (0.8)	< 0.001
Diseases of the eye & adnexa			
**Cataract, *n* (%)**	185 (0.7)	375 (0.7)	< 0.001
Diseases of the circulatory system			
**Hypertension, *n* (%)**	5,033 (18.2)	8,685 (15.4)	< 0.001
**Myocardial infarction, *n* (%)**	564 (2.0)	574 (1.0)	< 0.001
**Stroke, *n* (%)**	273 (1.0)	268 (0.5)	< 0.001
Diseases of the respiratory system			
**Asthma / Chronic bronchitis, *n* (%)**	161 (0.6)	318 (0.6)	0.616
Diseases of the digestive system			
**Cholelithiasis, *n* (%)**	34 (0.1)	63 (0.1)	0.888
**Fatty liver disease, *n* (%)**	302 (1.1)	222 (0.4)	< 0.001
**Gastritis, *n* (%)**	416 (1.5)	1,050 (1.9)	< 0.001
**Intestinal polyp, *n* (%)**	33 (0.1)	31 (0.1)	< 0.001
**Peptic Ulcer, *n* (%)**	285 (1.0)	464 (0.8)	0.012
Diseases of the musculo-skeletal system &connective tissue			
**Arthritis, *n* (%)**	350 (1.3)	2,871 (5.1)	< 0.001
**Osteoporosis, *n* (%)**	45 (0.2)	1,667 (3.0)	< 0.001
Diseases of the genitoruinary system			
**Bladder infection, *n* (%)**	33 (0.1)	225 (0.4)	< 0.001

a. *p*-value was calculated by chi-square test for categorical variable
