# Supplementary material for: Correlates of Self-Reported Sleep Duration in Middle-Aged and Elderly Koreans: from the Health Examinees Study
Source: PLoS One. 2015 May 1;10(5):e0123510. doi: 10.1371/journal.pone.0123510 (PMC4416918; doi:10.1371/journal.pone.0123510)
Supplement: S3 Table — (PDF) [file pone.0123510.s003.pdf]

S3 Table. Phase sensitivity analysis among men: odd ratios (95% CIs) [a] for short or long sleep duration compared to normal sleep duration (6-7 hours) according to selected correlates

	Sensitivity analysis I [a] (N=20,923)			Sensitivity analysis II [b] (N=20,731)			Sensitivity analysis III [c] (N=19,617)		
	< 6 hours OR (95% CI)	8 - 9 hours OR (95% CI)	≥ 10 hours OR (95% CI)	< 6 hours OR (95% CI)	8 - 9 hours OR (95% CI)	≥ 10 hours OR (95% CI)	< 6 hours OR (95% CI)	8 - 9 hours OR (95% CI)	≥ 10 hours OR (95% CI)
Sociodemographic factors									
**Age (years)**									
40-49	1.00 (Ref.)	1.00 (Ref.)	1.00 (Ref.)	1.00 (Ref.)	1.00 (Ref.)	1.00 (Ref.)	1.00 (Ref.)	1.00 (Ref.)	1.00 (Ref.)
50-59	1.07 (0.96-1.20)	1.09 (1.01-1.18)	1.61 (1.22-2.14)	1.08 (0.96-1.21)	1.09 (1.01-1.18)	1.63 (1.24-2.17)	1.09 (0.97-1.22)	1.08 (0.99-1.17)	1.68 (1.26-2.24)
60-69	1.65 (1.44-1.89)	1.06 (0.95-1.17)	1.96 (1.42-2.70)	1.65 (1.44-1.66)	1.06 (0.96-1.17)	2.01 (1.46-2.78)	1.61 (1.40-1.86)	1.03 (0.92-1.14)	2.00 (1.43-2.80)
**Educational attainment**									
College degree or higher	1.00 (Ref.)	1.00 (Ref.)	1.00 (Ref.)	1.00 (Ref.)	1.00 (Ref.)	1.00 (Ref.)	1.00 (Ref.)	1.00 (Ref.)	1.00 (Ref.)
High school graduate	1.12 (0.99-1.27)	1.30 (1.20-1.42)	1.67 (1.20-2.33)	1.13 (1.00-1.28)	1.30 (1.19-1.42)	1.65 (1.18-2.31)	1.12 (0.98-1.27)	1.31 (1.19-1.43)	1.71 (1.21-2.42)
Middle school or below	1.43 (1.24-1.65)	1.57 (1.42-1.75)	2.65 (1.87-3.77)	1.44 (1.24-1.66)	1.57 (1.42-1.75)	2.53 (1.78-3.61)	1.45 (1.25-1.68)	1.61 (1.44-1.79)	2.59 (1.79-3.73)
**Occupational classification**									
Non-manual	1.00 (Ref.)	1.00 (Ref.)	1.00 (Ref.)	1.00 (Ref.)	1.00 (Ref.)	1.00 (Ref.)	1.00 (Ref.)	1.00 (Ref.)	1.00 (Ref.)
Manual	1.14 (1.01-1.30)	1.15 (1.05-1.25)	1.59 (1.14-2.24)	1.14 (1.01-1.30)	1.15 (1.05-1.26)	1.61 (1.15-2.26)	1.15 (1.01-1.30)	1.14 (1.04-1.25)	1.68 (1.19-2.39)
Unemployed / housewives	1.02 (0.87-1.20)	1.44 (1.29-1.61)	2.39 (1.63-3.50)	1.02 (0.87-1.20)	1.45 (1.20-1.62)	2.32 (1.58-3.41)	1.02 (0.86-1.21)	1.44 (1.27-1.61)	2.38 (1.60-3.54)
**Marital status**									
Married	1.00 (Ref.)	1.00 (Ref.)	1.00 (Ref.)	1.00 (Ref.)	1.00 (Ref.)	1.00 (Ref.)	1.00 (Ref.)	1.00 (Ref.)	1.00 (Ref.)
Single or separated	1.23 (1.02-1.48)	0.86 (0.74-1.01)	0.96 (0.63-1.48)	1.23 (1.02-1.49)	0.86 (0.74-1.01)	0.96 (0.62-1.49)	1.25 (1.03-1.52)	0.90 (0.76-1.05)	0.87 (0.54-1.39)
**Menopausal status**									
Premenopausal	-	-	-	-	-	-	-	-	-
Postmenopausal	-	-	-	-	-	-	-	-	-
Lifestyle factors									
**Smoking status**									
Non-current smokers	1.00 (Ref.)	1.00 (Ref.)	1.00 (Ref.)	1.00 (Ref.)	1.00 (Ref.)	1.00 (Ref.)	1.00 (Ref.)	1.00 (Ref.)	1.00 (Ref.)
Current smokers	0.97 (0.88-1.08)	1.04 (0.97-1.12)	1.35 (1.08-1.69)	0.97 (0.88-1.08)	1.05 (0.97-1.13)	1.34 (1.07-1.68)	0.96 (0.86-1.07)	1.04 (0.96-1.12)	1.35 (1.07-1.70)
**Alcohol consumption**									
Non-current drinkers	1.00 (Ref.)	1.00 (Ref.)	1.00 (Ref.)	1.00 (Ref.)	1.00 (Ref.)	1.00 (Ref.)	1.00 (Ref.)	1.00 (Ref.)	1.00 (Ref.)
Current drinkers	0.90 (0.81-1.00)	0.96 (0.89-1.04)	1.05 (0.83-1.33)	0.90 (0.81-1.00)	0.96 (0.89-1.04)	1.09 (0.85-1.39)	0.90 (0.81-1.01)	0.98 (0.90-1.06)	1.07 (0.83-1.38)
**Eating habits**									
Having three meals a day	1.00 (Ref.)	1.00 (Ref.)	1.00 (Ref.)	1.00 (Ref.)	1.00 (Ref.)	1.00 (Ref.)	1.00 (Ref.)	1.00 (Ref.)	1.00 (Ref.)
Having irregular meals	1.51 (1.33-1.72)	0.99 (0.89-1.10)	1.94 (1.48-2.53)	1.50 (1.32-1.71)	0.99 (0.89-1.10)	1.99 (1.52-2.60)	1.51 (1.33-1.73)	0.97 (0.87-1.08)	1.97 (1.50-2.60)
**Intake of multi vitamin**									
Multi-vitamin users	1.00 (Ref.)	1.00 (Ref.)	1.00 (Ref.)	1.00 (Ref.)	1.00 (Ref.)	1.00 (Ref.)	1.00 (Ref.)	1.00 (Ref.)	1.00 (Ref.)
Non-users	1.02 (0.90-1.16)	0.95 (0.87-1.04)	1.51 (1.06-2.15)	1.02 (0.90-1.16)	0.95 (0.87-1.04)	1.47 (1.03-2.10)	1.01 (0.89-1.17)	0.96 (0.87-1.05)	1.45 (1.01-2.08)
**Physical activity**									
Regular exercisers	1.00 (Ref.)	1.00 (Ref.)	1.00 (Ref.)	1.00 (Ref.)	1.00 (Ref.)	1.00 (Ref.)	1.00 (Ref.)	1.00 (Ref.)	1.00 (Ref.)
Non-exercisers	1.07 (0.97-1.18)	1.05 (0.98-1.12)	1.68 (1.34-2.11)	1.06 (0.96-1.17)	1.05 (0.98-1.13)	1.72 (1.36-2.16)	1.05 (0.95-1.17)	1.05 (0.97-1.12)	1.67 (1.31-2.11)

Psychological condition									
**PWI status [e]**									
Positive wellbeing	1.00 (Ref.)	1.00 (Ref.)	1.00 (Ref.)	1.00 (Ref.)	1.00 (Ref.)	1.00 (Ref.)	1.00 (Ref.)	1.00 (Ref.)	1.00 (Ref.)
Moderate distress	0.91 (0.79-1.06)	0.89 (0.81-0.99)	0.73 (0.54-1.00)	0.91 (0.79-1.05)	0.89 (0.80-0.98)	0.74 (0.54-1.01)	0.92 (0.79-1.06)	0.89 (0.80-0.99)	0.74 (0.54-1.02)
Severe distress	1.28 (1.04-1.58)	0.99 (0.85-1.16)	0.93 (0.59-1.46)	1.28 (1.04-1.58)	0.98 (0.84-1.15)	0.89 (0.57-1.41)	1.31 (1.05-1.62)	1.03 (0.87-1.21)	0.89 (0.56-1.43)
**Stress events**									
Not at all	1.00 (Ref.)	1.00 (Ref.)	1.00 (Ref.)	1.00 (Ref.)	1.00 (Ref.)	1.00 (Ref.)	1.00 (Ref.)	1.00 (Ref.)	1.00 (Ref.)
Often	1.17 (1.05-1.30)	0.90 (0.84-0.97)	0.92 (0.72-1.17)	1.17 (1.05-1.30)	0.90 (0.84-0.97)	0.91 (0.71-1.17)	1.19 (1.07-1.33)	0.91 (0.84-0.98)	0.91 (0.71-1.18)
Frequent	1.77 (1.48-2.12)	0.93 (0.79-1.09)	1.16 (0.77-1.75)	1.76 (1.47-2.10)	0.93 (0.79-1.08)	1.17 (0.77-1.77)	1.70 (1.41-2.06)	0.92 (0.78-1.09)	1.09 (0.70-1.69)
**Self-reported health status**									
Healthy	1.00 (Ref.)	1.00 (Ref.)	1.00 (Ref.)	1.00 (Ref.)	1.00 (Ref.)	1.00 (Ref.)	1.00 (Ref.)	1.00 (Ref.)	1.00 (Ref.)
Normal	0.90 (0.81-1.00)	0.99 (0.92-1.07)	1.10 (0.86-1.41)	0.91 (0.81-1.01)	0.99 (0.92-1.07)	1.11 (0.87-1.42)	0.89 (0.80-1.00)	0.99 (0.91-1.07)	1.11 (0.87-1.43)
Unhealthy	1.26 (1.08-1.47)	1.07 (0.95-1.21)	1.47 (1.04-2.06)	1.28 (1.09-1.50)	1.07 (0.94-1.21)	1.45 (1.02-2.06)	1.24 (1.05-1.47)	1.04 (0.92-1.19)	1.49 (1.03-2.14)
Anthropometry									
**Body mass index [f] (kg/m^2)**									
Quartile 1 (Lowest)	1.00 (0.87-1.16)	1.05 (0.95-1.16)	0.78 (0.95-1.16)	1.00 (0.87-1.16)	1.04 (0.94-1.15)	0.76 (0.54-1.07)	1.01 (0.87-1.18)	1.04 (0.94-1.15)	0.76 (0.53-1.07)
Quartile 2	1.00 (Ref.)	1.00 (Ref.)	1.00 (Ref.)	1.00 (Ref.)	1.00 (Ref.)	1.00 (Ref.)	1.00 (Ref.)	1.00 (Ref.)	1.00 (Ref.)
Quartile 3	1.12 (0.97-1.30)	0.97 (0.88-1.07)	0.99 (0.73-1.34)	1.12 (0.97-1.29)	0.96 (0.87-1.06)	0.98 (0.72-1.33)	1.11 (0.96-1.29)	0.96 (0.86-1.06)	0.97 (0.71-1.33)
Quartile 4 (Highest)	1.39 (1.18-1.64)	0.88 (0.78-0.99)	0.68 (0.47-0.99)	1.39 (1.18-1.64)	0.87 (0.77-0.98)	0.68 (0.47-0.99)	1.38 (1.16-1.63)	0.87 (0.77-0.98)	0.71 (0.48-1.04)
**Waist circumference [g] (cm)**									
Quartile 1 (Lowest)	1.02 (0.89-1.18)	0.91 (0.82-1.00)	0.92 (0.65-1.30)	1.03 (0.89-1.19)	0.91 (0.82-1.00)	0.94 (0.67-1.34)	0.99 (0.85-1.15)	0.89 (0.81-0.99)	0.88 (0.62-1.26)
Quartile 2	1.00 (Ref.)	1.00 (Ref.)	1.00 (Ref.)	1.00 (Ref.)	1.00 (Ref.)	1.00 (Ref.)	1.00 (Ref.)	1.00 (Ref.)	1.00 (Ref.)
Quartile 3	0.88 (0.76-1.01)	0.94 (0.85-1.03)	1.08 (0.78-1.48)	0.88 (0.76-1.01)	0.93 (0.84-1.03)	1.11 (0.80-1.53)	0.89 (0.77-1.03)	0.93 (0.84-1.03)	1.03 (0.74-1.44)
Quartile 4 (Highest)	0.87 (0.73-1.03)	1.10 (0.98-1.24)	1.65 (1.15-2.37)	0.87 (0.73-1.03)	1.10 (0.97-1.24)	1.67 (1.15-2.41)	0.88 (0.74-1.05)	1.10 (0.98-1.25)	1.60 (1.09-2.32)

Note: Unknown values were wholly included in the statistical models but were not presented in the table

a. All ORs were adjusted for sociodemographic factors (*i.e.*, age, education attainment, occupational classification, marital status, and menopausal status only for women), lifestyle factors (*i.e.*, smoking status, alcohol consumption, eating habits, intake of multi vitamin, and physical activity), psychological condition (*i.e.*, PWI status, stress events, and self-reported health status), and anthropometry (*i.e.*, body mass index and waist circumference)

b. Subjects who were currently taking any medications for diabetes mellitus, depression, hypertension, stroke, peptic ulcer, and arthritis were excluded from the primary analysis

c. According to sex strata, men who were currently taking any medications for cancer, diabetes mellitus, thyroid disease, depression, hypertension, stroke, peptic ulcer, and arthritis were excluded from the primary analysis

d. Subjects who were currently taking any medications for selected diseases were wholly excluded from the primary analysis

e. Defined as positive wellbeing group with PWI score ≤ 8, moderate distress group with 8 < PWI score < 27, and severe distress group with PWI score ≥ 27

f. Defined as follows: Quartile 1: ≤ 22.6, Quartile 2: 22.6 < BMI ≤ 24.4, Quartile 3: 24.4 < BMI ≤ 26.1, and Quartile 4: 26.1< BMI in men

g. Defined as follows: Quartile 1: ≤ 81.3, Quartile 2: 81.3 < WC ≤ 86.0, Quartile 3: 86.0 < BMI ≤ 91.0, and Quartile 4: 91.0 < BMI in men
